# Dietary Polyphenol, Gut Microbiota, and Health Benefits

**DOI:** 10.3390/antiox11061212

**Published:** 2022-06-20

**Authors:** Xiaofei Wang, Yue Qi, Hao Zheng

**Affiliations:** National Engineering Technology Research Center for Fruit and Vegetable Processing, Key Open Laboratory of Fruit and Vegetable Processing, Ministry of Agriculture and Rural Affairs, Beijing Key Laboratory of Food Non-Thermal Processing, College of Food Science and Nutritional Engineering, China Agricultural University, Beijing 100083, China; xiaofei.wang@cau.edu.cn (X.W.); qiyue@cau.edu.cn (Y.Q.)

**Keywords:** dietary polyphenols, host health, gut microbiota, biotransformation

## Abstract

Polyphenols, which are probably the most important secondary metabolites produced by plants, have attracted tremendous attention due to their health-promoting effects, including their antioxidant, anti-inflammatory, antibacterial, anti-adipogenic, and neuro-protective activities, as well as health properties. However, due to their complicated structures and high molecular weights, a large proportion of dietary polyphenols remain unabsorbed along the gastrointestinal tract, while in the large intestine they are biotransformed into bioactive, low-molecular-weight phenolic metabolites through the residing gut microbiota. Dietary polyphenols can modulate the composition of intestinal microbes, and in turn, gut microbes catabolize polyphenols to release bioactive metabolites. To better investigate the health benefits of dietary polyphenols, this review provides a summary of their modulation through in vitro and in vivo evidence (animal models and humans), as well as their possible actions through intestinal barrier function and gut microbes. This review aims to provide a basis for better understanding the relationship between dietary polyphenols, gut microbiota, and host health.

## 1. Introduction

Polyphenols, described as plants’ secondary metabolites, are probably the most abundant antioxidants in our daily life. The main dietary sources of these compounds include fruits, vegetables, grains, green tea, coffee, etc. [1]. Total dietary polyphenol intake is as high as 1 g per day for each adult, which is about 10-times higher than the intake of Vitamin C, and even 100-times higher than that of Vitamin E and carotenoids [2]. During the last few decades, there has been tremendous research output related to the health-promoting effects of polyphenols, including their antioxidant, anti-inflammatory, antibacterial, anti-adipogenic, and neuro-protective activities [3,4]. 

It has been reported that most dietary polyphenol intake remains unabsorbed in the small intestine, while the unabsorbed parts may accumulate in the large intestine and are extensively metabolized by the gut microbiota [5]. Therefore, intestinal microbiota play an important role in the biotransformation and metabolism of the original polyphenolic structures into low-molecular-weight metabolites, which can be readily absorbed and contribute to host healthy benefits. However, little is currently known regarding the possible mechanism among dietary polyphenols, gut microbes, and host health.

Dietary polyphenols influence gut microbiota compositions in the host, which further affect the host’s metabolism. In turn, intestinal microbiota can metabolize polyphenols into bioactive, low-molecular-weight phenolic metabolites to modulate the regulatory metabolism network. In this regard, this review aims to provide an assessment of dietary polyphenols’ biological significances on host health, a summary of their modulation through in vitro and in vivo evidence (animal models and humans), as well as their possible action through intestinal barrier function and gut microbes.

## 2. Dietary Polyphenols and Their Sources

**Figure 1:**
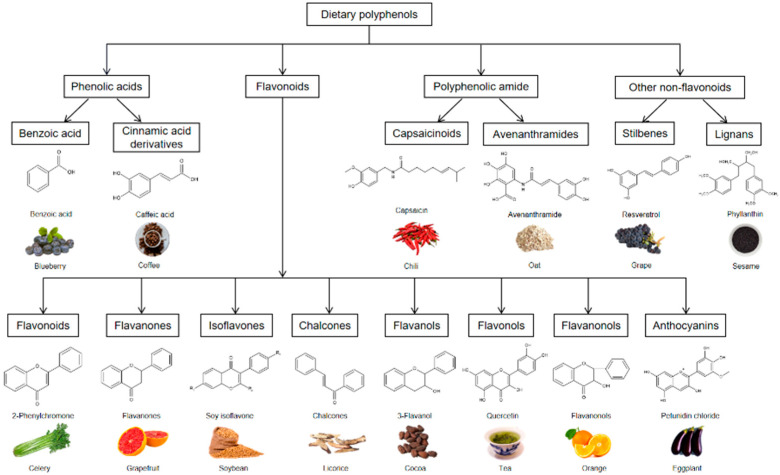
Classification of dietary polyphenols and their sources.

Dietary polyphenols are one of the most abundant and widely distributed natural products in plants. At present, according to the structure, dietary polyphenols are divided into four categories: phenolic acids, flavonoids (the largest subclass of polyphenols), polyphenolic amide, and other non-flavonoids (Figure 1). Phenolic acids can be further divided into two main types, benzoic acid and cinnamic acid derivatives based on C1–C6 and C3–C6 backbones [6]. Flavonoids include flavonoids, flavanones, isoflavones, chalcones, flavanols, flavonols, flavanonols, anthocyanins, and so on [7]. Polyphenolic amides have N-containing functional substituents, two such groups are capsaicinoids and avenanthramides. The non-flavonoids include mainly stilbenes and lignans. In addition to phenolic acids, flavonoids, and phenolic amides, there are several non-flavonoid polyphenols found in foods that are considered important to human health, such as resveratrol, ellagic acid and its derivatives, curcumin, etc. The remarkable feature of the chemical structure is that it has a different amount of phenolic hydroxyl groups, which can be divided into phenolic monomers and polymerized polyphenols. Phenolic monomers include flavonoids and non-flavonoids. The former generally involves a common carbon skeleton of diphenyl propane in which two benzene rings are connected by a linear three-carbon chain, while the latter is two benzene rings connected by the vinyl group [8]. Polymeric polyphenols are oligomers or polymers polymerized by monomers known as tannins.

Polyphenols are widely distributed in nature, including in fruits, vegetables, cereals, beans, tea, coffee, honey, and red wine, which are the main sources of dietary polyphenols. Specifically, caffeic acid and ferulic acid are the most common phenolic acids in food. Caffeic acid is abundant in vegetables, fruits, and coffee, while ferulic acid is mainly distributed in rice bran, wheat bran, and other cereals. Among the flavonols, quercetin is the most common, which is commonly found in onions. Flavanols or flavan-3-ols are often commonly called catechins, which are abundant in red wine, chocolate, and lotus root. Isoflavones are mainly found in the leguminous family of plants. Anthocyanidins in plants mainly exist in glycosidic forms, which are commonly referred to as anthocyanins [9] and are largely distributed in strawberries, blueberries, and cherries. Some polyphenols have N-containing functional substituents, such as capsaicinoids in chili peppers and avenanthramides in oats, which belong to polyphenolic amides. The second major non-flavonoid group mainly consists of stilbenes, with resveratrol being the main representative, which is found in red and purple grape skins and grape wine. Another important nonflavonoid group is the lignans, which exist in bound forms in flax, sesame, and many grains.

## 3. Dietary Polyphenols and Their Biological Significance

As the most general plant-derived bioactive components in our diet, dietary polyphenols have received tremendous attention among nutritionists, food researchers, and consumers. Phenolic compounds are generally involved in defenses against plant pathogens and atmospheric agents, including bacteria, fungi, and viruses, and many abiotic stresses like drought, salinity, and UV. Polyphenols exhibit antimicrobial and antioxidant properties that can help plants to evade pathogenic infections and, at the same time, protect the major tissues from the toxic effects of reactive oxygen species [10]. Currently, they represent a topic of great scientific attention due to interest in their biological significance for humans. Both in vitro and in vivo studies have shown their health-promoting effects, including their antioxidant, anti-inflammatory, antibacterial, anti-adipogenic, and neuro-protective activities. 

### 3.1. Antioxidant Properties

The effectiveness of phenolic compounds in the inhibition of oxidative processes is potentially related to their reactive species scavenging activity. Due to the structure of the hydroxyl group on the benzene ring, polyphenols scavenge free radicals by H-atom transfer from the active OH group of the polyphenol to the free radical [6]. This allows polyphenols to indirectly activate antioxidant responses and generate non-toxic levels of intermediates, specifically the electrophilic forms of hydroquinone and quinone [11]. On the other hand, polyphenols inhibit the formation of or deactivation of the active species and precursors of free radicals, thus reducing the rate of oxidation and ultimately suppressing the generation of free radicals. They donate an electron to the free radical, neutralizing the radicals, and causing themselves to become stable (less reactive) radicals, thus stopping the reactions [12]. Treatment of HepG2 cells with (−)-epigallocatechin-3-gallate from green tea stimulates the nuclear translocation of nuclear factor erythroid 2-related factor 2 (Nrf2), which modulates the expression of antioxidant genes [13]. Resveratrol improves antioxidant defenses in pancreatic tissue because it enhances the activity of antioxidant enzymes such as catalase (CAT), superoxide dismutase (SOD), glutathione peroxidase (GPx), and glutathione-S-transferase (GST) [14]. 

### 3.2. Anti-Inflammatory Properties

Oxidative-stress-induced inflammation is mediated by the activation of cellular signaling processes of nuclear factor-kappa B (NF-kB) activation and activator protein-1 (AP-1) DNA binding [15]. It affects the expression of pro-inflammatory genes such as interleukin-1beta (IL-1β), IL-6, tumor necrotic factor alpha (TNF-a), and inducible nitric oxide synthase (iNOS) [16]. Preclinical and clinical studies suggest that polyphenols are able to express anti-inflammatory properties [17]. Although the precise mechanisms deserve further clarification, dietary polyphenols have shown benefits in distinct disorders [18]. Dihydroxylated phenolic acids produced from dietary proanthocyanidins potentially lowered the secretion of cytokines, including TNF-α, IL-1β, and IL-6, from healthy individuals [19]. Supplementation with 0.8% quercetin decreased interferon-γ, IL-1α, and IL-4 in male C57Bl/6j mice [20]. The administration of 10 mg/kg of quercetin also reduced the plasma nitrate plus nitrite (NOx) concentration and TNF-α production in adipose tissue of obese Zucker rats, resulting in an important anti-inflammatory effect [21].

### 3.3. Antibacterial Properties

Dietary polyphenols and plants rich in polyphenols have been demonstrated to be natural antimicrobials against both Gram-positive and Gram-negative bacteria. Epigallocatechin gallate (EGCG) was able to bind directly to the peptidoglycan from *Staphylococcus aureus*, affecting its cell integrity and destroying the osmotic protection of the cell wall [22]. Other than bacterial cell walls, tea polyphenols also damaged the inner cytoplasmic membrane of *Serratia marcescens*, increasing its permeability and releasing small cellular molecules [23]. Moreover, polyphenols can exhibit antibacterial activity via anti-biofilm agents. Cranberry proanthocyanidins limited the motility—particularly swarming motility—and reduced the biofilm formation of *Pseudomonas aeruginosa* [24]. However, due to the structural diversity of polyphenol classes, the mechanisms of their antimicrobial activities have not yet been fully resolved. 

### 3.4. Anti-Adipogenic Properties

Stimulating the development of beige adipocytes (so called ‘browning’) can reduce adverse obesity effects and help to improve metabolic health [25,26]. Dietary polyphenols have been demonstrated to effectively activate adipose tissue browning and relieve obesity and lipid accumulation through the induction of beige adipocytes. Daily ingestion of a catechin-rich beverage increases brown adipose tissue density in healthy young women, supporting the brown adipogenesis of polyphenols [27]. Also, in mice fed with a high energy diet, vanillic acid could accelerate thermogenesis and mitochondrial synthesis in both classical brown adipose tissue (BAT) and inguinal white adipose tissue (WAT) [28]. Resveratrol decreased triglycerides (TG) accumulation in the liver by suppressing the expression of adipogenesis-related genes, such as acetyl-CoA carboxylase (ACC), peroxisome proliferator-activated receptor (PPAR-γ), and sterol regulatory element binding protein (SREBP-1) [26,29]. Piceatannol treatment suppressed protein levels of the adipogenic transcription factors PPAR-γ, while it increased ACC protein expression [30]. Therefore, a positive relationship may exist between dietary polyphenol and anti-adipogenesis, and the underlying mechanisms are worthy of exploration.

### 3.5. Neuro-Protective Properties

The neuro-protective effects of dietary polyphenols have received considerable attention in recent years, suggesting that polyphenols may be effective in reversing neurodegenerative pathology and age-related declines in neurocognitive performance. Animal evidence demonstrates that blueberries are effective at reversing age-related deficits in rat spatial working memory, and (−)-epicatechin enhances the retention of mice spatial memory and may relate to their potential to influence the synthesis of neurotrophic factors [31,32]. In addition, curcumin could disrupt existing plaques and partially restore distorted neurites in an Alzheimer mouse model [33]. Resveratrol can activate the phosphorylation of protein kinase C and secretes transthyretin to prevent Aβ aggregation in cultured rat hippocampal cells [34]. However, a direct association between dietary polyphenol and an improvement in neurological health has not been made at present.

## 4. Impact of Dietary Polyphenols on Gut Microbiota

Emerging evidence demonstrates that gut microbiota plays an important role in maintaining the physiological function of host health and the pathogenesis of various diseases, including obesity, diabetes, inflammatory bowel disease, and even neurodegenerative disorders. Diet can alter the composition of gut microbiota, which in turn affects host metabolism. The alteration of gut microbiota by the administration of probiotics, prebiotics, or fecal microbiota transplantation is already well established. However, the gut microbiota-modulating effects of polyphenol are less clear. Nevertheless, there is growing evidence showing that dietary polyphenol may directly modulate the gut microbiome, i.e., increasing beneficial microbial or decreasing harmful microbial species in the gut microbiota. In this part, we summarize the in vitro and in vivo studies that studied the effects of polyphenol supplementation on the gut microbiota.

### 4.1. In Vitro Modulation of Dietary Polyphenols on Gut Microbiota 

In vitro experiments on polyphenols and polyphenol-rich food sources have been studied through extraction, digestion, and fermentation to demonstrate that they could modulate the resident bacteria. A series of in vitro studies with polyphenol from different sources have been listed in Table 1, including grapes, berries, tea, pomegranate, and other plants, to demonstrate the regulatory effect of polyphenol supplementation on intestinal micro-organisms. 

Polyphenols can selectively inhibit the growth of pathogenic bacteria. Flavonoids in red wine showed a slight inhibition of the *Clostridium* [35]. Ellagic acid and anthocyanins in raspberry juice may inhibit the growth of *Ruminococcus* [37]. Grape polyphenols can inhibit the growth of *Clostridium histolyticum* [36]. On the other hand, polyphenols can promote the growth of beneficial bacteria in the gut, such as *Bifidobacterium*. Tannin in pomegranate, gingerol in ginger, grape polyphenols, and sorghum polyphenols can promote the growth of *Bifidobacterium* [41,45,46]. Tannin can also promote the growth of *Lactobacillus* [45]. Gingerol and grape polyphenols can promote the growth of *Enterococci* [36,45]. Sorghum polyphenols can cooperate with fructooligosaccharides to enhance the abundance of lactic acid bacteria, *Roseburia,* and *Prevotella* [46]. However, Kemperman’s research shows that polyphenols in red wine and black tea can reduce the abundance of *Bifidobacterium* [39]. They conducted in vitro experiments using fluids from the colon and found that catechins and flavonoids in black tea could stimulate *Klebsiella, Enterococci,* and *Akkermansia* and reduce *Bifidobacteria*, *B. coccoids, Anaeroglobus,* and *Victivallis*. Anthocyanins and catechins in red wine can promote the growth of *Klebsiella, Alistipes, Cloacibacillus, Victivallis,* and *Akkermansia*, and reduce the growth of *Bifidobacteria, B. coccoides, Anaeroglobus, Subdoligranulum,* and *Bacteroides* [39]. Mango peel is another high-polyphenol food, with gallates, flavonoids, gallotannins, gallic acid, and so on, and in vitro fermentation of mango peel could increase the growth of *Bifidobacterium* and *Lactobacillus*. 

### 4.2. In Vivo Modulation of Dietary Polyphenols on Gut Microbiota of Animal Models

Similarly, in vivo studies have shown that polyphenol supplementation can modulate gut microbiota in animal models, including the increase of beneficial microbes and the decrease of harmful microbes. Detailed information on the published in vivo studies, from invertebrate *Drosophila* and zebrafish to vertebrate rat, mouse, chick, pig, etc., have been listed in Table 2. Both vertebrate and invertebrate model organisms confirmed that polyphenol supplementation can increase the number of beneficial bacteria in the gut, such as *Bifidobacterium* and *Lactobacillus*. Mango supplementation in mice fed with a high-fat diet can prevent the loss of beneficial intestinal bacteria, especially *Bifidobacteria, Akkermansia,* and *Aldercrutzia* [47]. Orso applied a diet of chestnut shell extract rich in tannin to a zebrafish intestinal inflammation model and found that it promoted the growth of healthy and beneficial bacteria (*Enterobacteriaceae* and *Pseudomonas*) [48]. Supplementation with polyphenols can also change the ratio of *Firmicutes* to *Bacteroides*. Cranberry extract is rich in phenolic acids, flavonoids, anthocyanins, and other polyphenols, which can reduce the ratio of *Firmicutes* to *Bacteroides* in mice induced by a high-fat/high-sugar diet [49]. Moreover, a polyphenol diet intervention can selectively inhibit pathogenic bacteria. Polyphenols from *Smilax china L. rhizome* can reduce the relative abundance of *Desulfovibrionaceae, Lachnospiraceae,* and *Streptococcaceae* [50], and grape pomace reduces potentially pathogenic bacteria to humans, such as *Salmonella, E. coli, Shigella, Yersinia,* and *Proteus* [51]. The combination of quercetin and resveratrol can significantly inhibit the relative abundance of *Desulfovibrionaceae, Acidaminococcaceae, Coriobacteriaceae, Bilophila,* and *Lachnospiraceae*, which may be related to diet-induced obesity [52]. Blueberry polyphenols were used to interfere with ovariectomized rats, with an upregulation of *Bacteroides dorei* and *Lachnoclostridium* and a decrease of *Rickenellaceae* and *Eubacterium* [53].

### 4.3. In Vivo Modulation of Dietary Polyphenols on Gut Microbiota of Humans

Clinical studies further confirmed the regulatory effect of polyphenols on human intestinal micro-organisms (Table 3). Consistent with in vitro and in vivo animal studies, supplementation with polyphenols such as anthocyanins and flavonoids increase the abundance of *Bifidobacterium* and *Lactobacillus*, which are two intestinal protective agents in the human gut [90,91]. Blueberries are rich in anthocyanins, which can increase the number of *Bifidobacteria* and lactic acid bacteria in healthy volunteers [92]. Almonds and almond skins are heavily rich in a range of flavonoids, including catechin, flavonol, and flavanone glycosides, and adding almonds or almond skins to the diet can increase the number of *Bifidobacteria* and *Lactobacillus* in feces [93]; Moreno-Indias found that polyphenols in red wine can increase the number of *Bifidobacteria* and *Lactobacillus* [94]. Besides, a diet rich in polyphenols can regulate the ratio of *Firmicutes* to *Bacteroides* in the human body. Daily consumption of cranberries rich in proanthocyanidins can reduce the number of *Firmicutes* in the body and increase the number of *Bacteroides* [95]; however, Yuan used tea polyphenols in tea to intervene in healthy volunteers and found different results. The diet that intervened with tea polyphenols resulted in an increase in the number of *Firmicutes* in feces, a decrease in the number of *Bacteroides*, and an increase in the ratio of *Firmicutes* to *Bacteroides* [96]. Queipo-Ortu found that the combined action of alcohol and polyphenols could increase the number of *Enterococcus, Prevotella, Bacteroides, Bifidobacterium*, *Bacteroides uniformis*, *Eggerthella lenta*, and *Blautia coccoides–Eubacterium*, but had no significant effect on the changes of *Lactobacillus* [97].

The effect of polyphenols on gut microbiota is related to the number of initial microbiota in the intestinal tract. Mayta-Apaza classified them according to the initial number of *Bacteroides* in the body, and different microbial compositions led to different performances after receiving a dietary intervention. After receiving sour cherry juice, the volunteers with high initial *Bacteroides* reduced *Bacteroides* and *Bifidobacterium* and increased the *Lachnospiraceae, Ruminococcus,* and potential polyphenol metabolite *Collinsella*. The volunteers with low *Bacteroides* responded to the increase of *Bacteroides* and *Bifidobacterium* and the decrease in the relative abundance of *Lachnospiraceae, Ruminococcus,* and *Collinsella* [38]. The effect of polyphenols on gut microbiota is related to the intake of polyphenols. Tzounis found that high-dose cocoa flavanone beverages increase the number of *Bifidobacterium*, lactic acid bacteria, and *Enterococci*; increase the number of *E.rectale–C.coccoides*; and reduce the number of *Histolytic Chlamydia*. A low dose of cocoa flavanone beverage will not cause a significant change in the number of *Bifidobacteria,* but will increase *Clostridia* [91].

## 5. Mechanism of Dietary Polyphenol and Gut Microbiota Affecting Host Health 

**Figure 2:**
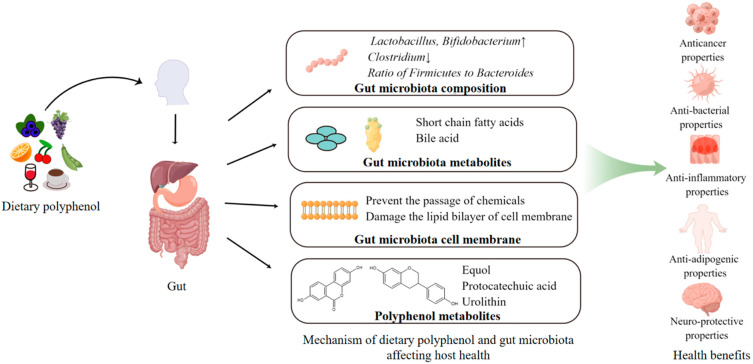
Possible mechanisms among dietary polyphenols, gut microbiota, and host health.

The gut microbiota and the host maintain normal physiological function and morphology of the intestine by forming a mutually beneficial relationship. Gut microbiota not only play a bridge role between the diet and host in digesting dietary food complexes, but also yields short-chain fatty acids and other metabolites to regulate human health. Studies have shown that only a small portion of polyphenols (5–10% of the total polyphenol intake) are absorbed in the small intestine, while most (90–95% of the total polyphenol intake) are transported to the human large intestine [104]. Diet polyphenol can modulate the gut microbial composition, and, at the same time, gut microbiota also improve the bioavailability of polyphenols by converting them to bioavailable metabolites (Figure 2).

### 5.1. Dietary Polyphenols Affect the Composition of Gut Microbiota

Dietary polyphenol has a definite role in the composition and functional profile of the gut microbiota. Polyphenols promote the growth of beneficial microbes, such as *Lactobacillus* and *Bifidobacterium*, which are two major health beneficial probiotics and bring benefits to human health, such as improving gastrointestinal disorders, suppressing diarrhea and constipation [105], alleviating lactose intolerance [106], relieving irritable bowel symptoms [107], and preventing inflammatory bowel disease [108]. A systematic review by Ma et al. with a meta-analysis revealed that polyphenol supplementation profoundly increased the abundance of *Lactobacillus* by 220% and *Bifidobacterium* by 56%. On the other hand, polyphenols can inhibit the growth of harmful microbiota, and *Clostridium histolyticum* and *Clostridium perfringens* in *Clostridium* are common pathogenic bacteria. *Clostridium histolyticum* causes inflammatory bowel disease [5] and *Clostridium perfringens* produces many toxins and hydrolytic enzymes, which are related to gastrointestinal disease and necrotizing enteritis [109]. Ma’s review system by meta-analysis showed that polyphenols derived from different foods all suppress the abundance of *Clostridium pathogen* species in the human gut microbiota, with tea being the most effective polyphenol food source for reducing *Clostridium* [110]. Dietary polyphenols can also regulate the ratio of *Firmicutes* to *Bacteroides*, which is related to body weight, and the ratio of *Firmicutes* to *Bacteroides* in obese patients is higher [111]. Xue’s studies have shown that four dietary polyphenols, rutin, quercetin, chlorogenic acid, and caffeic acid, can reduce the ratio of *Firmicutes* to *Bacteroides* in in vitro gut microbiota experiments [112]. However, due to the different types of polyphenols, polyphenol dosage, and research methods, the results of different studies are different to some extent, resulting in the changes between microbes not being completely consistent.

### 5.2. Dietary Polyphenols Affect the Metabolites of Gut Microbiota

Short-chain fatty acids (SCFAs) are the most well-studied microbial metabolites so far. SCFAs are a saturated aliphatic organic acid [113] that are produced by the incomplete metabolism of plant-derived carbohydrates by intestinal flora present in an anaerobic environment [114]. Acetate, propionate, and butyrate are the main SCFAs in the gut (accounting for 90% of the total SCFAs) [115]. Wu’s studies have shown that EGCG can significantly increase the number of SCFAs-producing bacteria, especially *Akkermansia*, and then promote the production of SCFAs, thereby enhancing anti-inflammatory effects and colon barrier integrity, which reduces enteritis [116]. Previous studies have shown that *Akkermansia muciniphila* can promote the production of acetate and propionate, and the nutritional interaction between *Akkermansia muciniphila* and butyrate-producing bacteria promotes butyrate production [117]. Liu’s experiment showed that after a week-long intervention with an Aronia-berry-rich diet, the polyphenol diet extracted by Aronia berry was 57% higher than that in the control group [3]. In the human model intestinal system, the in vitro fermentation of wild cherry juice increased the microbial production of propionate and butyrate [118]. McDougall found that after ingesting anthocyanin-rich raspberry, the concentration of bile acid in an ideal fluid of ileostomy subjects changed significantly, wherein the glycine and taurine derivatives of cholate and deoxycholate increased [119]. Fotschki further described the beneficial effects of raspberry dregs on the bile acid profile of the cecum in a hyperlipidemic mouse model [120]. Studies by Huang have shown that EGCG can significantly reduce the content of intestinal bile acid; increase the excretion of bile acid, cholesterol, and total lipids in feces; and alleviate metabolic abnormalities and fatty liver induced by a high-fat diet in mice [121]. Therefore, after dietary polyphenols reach the gut, microbiota can then further produce metabolites, and, once absorbed and transported to target tissues and organs, contribute to metabolite health. 

### 5.3. Dietary Polyphenols Affect the Bacterial Cell Membrane

Dietary polyphenol can interfere with the bacterial cell function of the cell membrane. For example, flavonols and flavones in the *Staphylococcus* genus can increase membrane cytoplasm permeability. Studies have shown that the antibacterial effect of polyphenols is more effective against Gram-positive bacteria. Inouye pointed out that because of the hydrophilic outer membrane outside the cell wall of Gram-negative bacteria, the passage of chemicals is prevented. Gram-negative bacteria are more resistant to plant secondary metabolites, including phenols [122]. When polyphenols were ingested, the growth of Gram-negative *Salmonella* and *Escherichia* strains was inhibited, but the growth of Gram-positive lactic acid bacteria was not affected [123]. The effect of polyphenols on bacteria depends on the interaction between compounds and the bacterial cell surface, which can inhibit bacterial growth by disturbing the function of the cell membrane [124]. Tea polyphenols, such as tea catechins, have a strong affinity to the lipid bilayers of the cell membrane through hydrogen bonds with the bilayer surface, thus penetrating underneath the surface and giving play to antibacterial, anticancer, and other beneficial effects [125]. EGCG has antibacterial activity against Staphylococcus; possible mechanisms include damaging the lipid bilayer of the cell membrane, reducing mucus production and affecting the formation of biofilm, and binding and neutralizing with enterotoxin B [126]. Therefore, the effect of polyphenols on the bacterial cell membranes is considered to be one of the mechanisms for regulating metabolic health.

### 5.4. Biotransformation of Polyphenols by Gut Microbiota

With respect to the complicated structures and high molecular weights, dietary polyphenols have low bioavailability and are difficult to be absorbed in the small intestine. About 90% of dietary polyphenols arrive at the colon in an intact form where they are biotransformed and metabolized into bioactive, low-molecular-weight phenolic metabolites through the residing microbiota [127]. Chen discovered that gut bacteria can deconjugate mulberry anthocyanin (cyanidin-3-glucoside, cyanidin-3-rutin, and delphinidin-3-rutinoside) to lower molecular-weight metabolites, and metabonomic data showed that the first two compounds were decomposed into protocatechuic, vanillic acid, and p-coumaric acids, while the latter was converted to syringic acid and gallic acid [128,129]. The core bacteria that can metabolize anthocyanins are *Bifidobacterium* spp. and *Lactobacillus* spp. [130,131] with probiotic effects to produce antibacterial substances, to compete with pathogens for adhering to the epithelium and for nutrients, to regulate the host immune system, and to inhibit the production of bacterial toxins [132]. The flavonoids (flavonols, flavones, and flavanones) can be biotransformed into p-hydroxyphenylacetic acid, protocatechuic acid, p-hydroxybenzoic acid, vanillic acid, hydrocaffeic acid, coumaric acid, 3-(4-hydroxyphenyl) propionic acid, and other aromatic metabolites [133]. Soybean isoflavones can be converted to dihydrodaidzein, dihydrogenistein,6′-OH-*O*-desmethylangolensin, and cis-4-OH-equol by anaerobic bacteria in the distal region of the small intestine and colon [134,135,136]. The bioavailability of ellagic tannin, which was found in pomegranate and grape, is low, but they can be metabolized by intestinal micro-organisms into urolithins with antioxidant activity and preventive effects for chronic diseases such as cancer, diabetes, and cardiovascular and neurodegenerative diseases [137,138]. Therefore, polyphenol metabolites produced by gut microbiota have potentially beneficial effects on the host. 

## 6. Conclusions

There is increasing evidence in the literature to emphasize that dietary polyphenols have potentially beneficial effects on host health through interactions with gut microbiota. Numerous studies listed in this review, both in vitro and in vivo, demonstrated the relationship between dietary polyphenols and gut microbiota, while the possible mechanism may be through the alteration of gut microbiota composition, the production of gut microbiota metabolites, the modulation of intestinal barrier function, and the biotransformation and metabolism of dietary polyphenols. However, a clear and deep understanding of these mechanisms between polyphenols and gut microbiota is necessitated, especially considering the metabolic pathways, which will allow for new therapeutic targets in the future. 

## Figures and Tables

**Table 1:** Study on the effect of polyphenols on gut microbiota in vitro.

Polyphenol and Source	Model	Impact on Microbiota	Reference
Flavonoids, Red wine	In vitro feces fermentation	Inhibit *Clostridium histolyticum* group	[35]
Grape polyphenol, Grape seeds	In vitro feces fermentation	Increase *Bifidobacterium* spp. and *Lactobacillus-Enterococcus* group; Inhibit *Clostridium histolyticum* group and the *Bacteroides-Prevotella* group	[36]
Ellagic acid and anthocyanins, Raspberry	In vitro colonic fermentation	Increase the abundance of *Escherichia coli*, butyric acid-producing bacteria, *Lactobacillus* and *Akkermansia*; Decrease *Bacteroides* and *Ruminococcus*.	[37]
Anthocyanins, flavonoids, neochlorogenic acids, tart cherry	The Simulator of the Human Intestinal Microbial Ecosystem	Increase *Bacteroidetes, Firmicutes, Proteobacteria* Decrease *Verrumicrobia*	[38]
Catechins and Flavonol, Black tea	The Simulator of the Human Intestinal Microbial Ecosystem	Increase *Klebsiella, enterococci, Akkermansia.* Reduce *bifidobacteria, B. coccoides, Anaeroglobus, Victivallis*	[39]
Green tea, oolong tea and black tea	In vitro fermentation Intestinal absorption	Increase *Bifidobacterium* spp., *Lactobacillus*/*Enterococcus* spp.; Decrease *Firmicutes*/*Bacteroidetes* ratio and *Clostridium histolyticum*	[40]
Ellagitannins, Pomegranate by-product	In vitro feces fermentation	Enhance *Bifidobacterium* spp. and *Lactobacillus* spp.	[41]
Mango peel	In vitro model of the colon	Enhance *Bifidobacterium* and *Lactobacillus*	[42]
Red fruit	In vitro fermentation	Decrease *B. cereus*, *S. aureus*, *E. coli*	[43]
Olive pomace	In vitro feces fermentation	Increase *Firmicutes* and *Bacteroidetes* groups	[44]
6-gingerols, Ginger	Simulated digestion model in vitro	Increase *Bifidobacterium* and *Enterococcus*	[45]
Proanthocyanidins, Sorghum bran	In vitro model of the colon	Increase *Bifidobacterium* spp., *Lactobacillus–Enterococcus* group; Decrease *Clostridium histolyticum* group, *Bacteroides–Prevotella* group	[46]

**Table 2:** Effect of polyphenols on animal gut microbiota.

Polyphenol and Source	Model	Impact on Microbiota	Reference
	Rat		
Epicatechin and catechin, Commercial	Wistar rats	Decrease *Bacteroides, Clostridium and Staphylococcus*	[54]
Quercetin and Resveratrol, Commercial	HFD (High-fat-diet) rats	Reduce *Firmicutes* and the proportion of *Firmicutes* to *Bacteroidetes*.	[52]
Sinapic acid and resveratrol, Commercial	HFD rats	Increase *Lachaospiraceae*; Decrease *Bacteroides and Desulfovibrionaceaesp*	[55]
Chlorogenic acid, Commercial	Wistar male rats	Increase *Burkholderiales, Bifidobacterium*; Decrease *Desulfovibrionales, Desulfovibrio, Klebsiella,*	[56]
Hesperetin, Commercial	Rats	Increase *Bifidobacterium, Lactobacillales*; Decrease *Clostridium subcluster XIVa*	[57]
Blueberry polyphenols, Blueberry	Rats	Reduce the *Firmicutes* to *Bacteroidetes* ratio; Increase *Proteobacteria Bacteroides dorei* and *Lachnoclostridium*.	[53]
Epicatechin and procyanidin, Cocoa	Male Zucker diabetic fatty rats	Increase acetate-producing bacteria such as *Blautia*; Prevent lactate-producing bacteria (*Enterococcus* and *Lactobacillus genera*)	[58]
Gallic acid	Rats	Increase *Lactobacillus, Bifidobacterium, Enterobacteriaceae*	[59]
Pomegranate peel	HFD rats	Decrease *Firmicutes* to *Bacteroidetes* ratio; Increase *Bacteroidales, Lactobacillus*	[60]
Persimmon tannin	Rats	Decrease *Firmicutes/Bacteroidetes* ratio; Increase *Bifidobacterium* spp., *Lactobacillus* spp	[61]
Seaweed polyphenols	HFD/streptozotocin rats	Increase *Odoribacter, Muribaculum, Parabacteroides*; Decrease *Firmicutes/Bacteroidetes* ratio	[62]
Phenolic acids, flavan-3-ols	A high salt diet fed rats	Increase *Bacteroidetes, Ruminococcaceae*; Decrease *Proteobacteria, Erysipelotrichaceae*	[63]
Ellagic acid, gallic acid, and quercetin-3-rutinoside	Colon cancer rats	Increase *Bacteroidetes*; Decrease *Firmicutes*	[64]
	Mice		
Resveratrol, Commercial	HFD mice	Increase *Bacteroidetes*; Decrease *Firmicutes*	[65]
Chlorogenic acid, Commercial	HFD mice	Increase *Bacteroidaceae, Lactobacillaceae*; Decrease *Desulfovibrionaceae, Ruminococcaceae, Lachnospiraceae*	[66]
Tea polyphenols, Commercial	HFD mice	Increase *Actinobacteria*; Decrease *Proteobacteria*	[67]
Anthocyanins, Commercial	Mice	Increase *Lachnospiraceae*; Decrease *Bacilli, Clostridia*	[3]
Flavonoid apigenin, Commercial	Mice	Increase *Actinobacteria*; Decrease *Firmicutes*	[68]
Phenolic acids, flavonoids, anthocyanins, Cranberry	High fat/sucrose mice	Reduce the *Firmicutes* to *Bacteroidetes* ratio; Stimulate the growth of *Akkermansia* spp.	[49]
Caffeoylquinic acid, Quercetin, *Smilax china L. rhizome*	High fat/high sucrose mice	Decrease ratios of *Firmicutes* to *Bacteroidetes*; Increase *Desulfovibrionaceae*, *Streptococcaceae*, *Akkermansiaceae*	[50]
Betacyanins, Red pitayas	HFD mice	Decrease the ratio of *Firmicutes* to *Bacteroidetes*; Increase the relative abundance of *Akkermansia.*	[69]
Flavonoids, Painong-San	Colitis mice	Increase *Romboutsia, Lactobacillus, Bifidobacterium, Akkermansia*; Decrease *Oscillospiraceae, Helicobacter*	[70]
Gallic acid, *Canarium album*	HFD mice	Increase *Firmicutes, Verrucomicrobia, Akkermansia*; Decrease of *Bacteroidetes*	[71]
Gallic acid, anthocyanins, epicatechin, Berry	High-fat/sucrose mice	Increase *Akkermansiaceae*; Decrease *Firmicutes, Lachnospiraceae, Ruminococcaceae, Peptostreptococcaceae*	[72]
Flavonoid, Penthorum chinense pursh	Mice	Increase *Bacteroidetes, Proteobacteria, Verrucomicrobia*; Decrease *Firmicutes, Actinobacteria, Deferribacteres*	[73]
Grape polyphenols, Grape	Mice	Increase *Akkermansia,* *Lactobacillus*	[74]
Anthocyanins, Lycium ruthenicum Murray	Mice	Increase *Barnesiella,* *Alistipes,* *Eisenbergiella,* *Coprobacter,* *Odoribacter*	[75]
*Camellia japonica* bee pollen kaempferol	Oxonate-induced mice	Increase *Firmicutes*; Decrease *Bacteroidetes,* *Actinobacteria,* *Proteobacteria*	[76]
Ellagitannins, ellagic acid, anthocyanins, Raspberry	Mice	Increase *Lactobacillus*; Decrease *Blautia, Ruminiclostridium*	[37]
Anthocyanidins, Lycium ruthenicum	Mice	Increase *Verrucomicrobia, Bacteroidetes, Akkermansia, Odoribacter, Bifidobacterium*; Decrease *Firmicutes*	[77]
Tea polyphenol, Kombucha	HFD/streptozotocin mice	Increase *Lactobacillus, Butyricicoccus*; Decrease *Proteobacteria, Desulfovibrio, Escherichia-Shigella, Bacteroidetes*	[78]
3-hydroxybenzylhydrazine, isophorone, Millet shells	HFD mice	Increase *Bacteroidetes*; Decrease *Verrucomicrobia, Actinobacteria*	[79]
Tea polyphenol, Tea extract	Colitis Mice	Increase *Faecalibaculum, Bifidobacterium*; Decrease *Bacteroids, Mucispirillum*	[80]
Mango Polyphenols, Mango pulp	HFD mice	Prevent the loss of beneficial gut bacteria, specifically *Bifidobacteria, Akkermansia,* and *Aldercrutzia*.	[47]
Chlorogenic acid, Chicory root	Mice	Increase *Prevotellaceae*, *Lachnospiraceae bacterium* A2, *Clostridium* ASF356, Decrease *Oscillospirales*, *Ruminococcus*, the ratio *Firmicutes*/*Bacteroidetes*	[81]
	Pig		
Gallic acid, ethyl gallate, Red-osier dogwood	Pig	Increase class *Bacilli*, *Lactobacillales* and family *lactobacillaceae*	[82]
Proanthocyanidin, Grape seed	Pig	Increase *Lachnospiraceae, Clostridales, Lactobacillus* and *Ruminococcacceae.*	[83]
Chlorogenic acid, Commercial	Pig	Increase *Lactobacillus* spp., *Prevotella* spp., *Anaerovibrio* spp., *and Alloprevotella* spp.; Decrease *Proteobacteria*	[84]
	Chick		
Procyanidins and anthocyanidins, Grape	Broiler chicks	Increase the populations of *Enterococcus*, *Escherichia coli, Lactobacillus*; Decrease the counts of *Clostridium.*	[85]
Pentagalloyl glucose, Eucalyptus	Broiler chicks	Increase the *Firmicutes* to *Bacteroidetes* ratio, *Verrucomicrobia*; Decrease *Actinobacteria, Proteobacteria*	[86]
Epicatechin and quercetin 3-glucoside, Carioca Bean	Broiler chicks	Increase *Coriobacteriaceae, Dehalobacteriaceae, Lachnospiraceae*	[87]
	Lamb		
Resveratrol, catechin, epicatechin, procyanidins, Grape pomace	Lambs	Enhance the growth of facultative probiotic bacteria and inhibit the growth of pathogen populations such as *Enterobacteriaceae* and *E. coli.*	[51]
	Zebrafish		
Tannins, Chestnut shells	Inflammation zebrafish	Increase the *Enterobacteriaceae, Pseudomonas* spp. and anaerobic bacteria (e.g., *Lactobacilli* and *Bifidobacteria*)	[48]
Dendrobium candidum	Inflammation zebrafish	Increase *Lactobacillus, Faecalibacterium, Rummeliibacillus*; Decrease *Shewanella, Geodermatophilus*	[88]
	*Drosophila*		
Eigallocatechin-3-gallate (EGCG), commercial	Rotenone-treated flies	Decrease *Proteobacteria,* *Acetobacter,* *Lactobacillus*; Increase the relative abundance of *Firmicutes* and *Bacteroidetes*	[89]

**Table 3:** Effect of polyphenols on human gut microbiota.

Polyphenol and Source	Impact on Microbiota	Reference
Anthocyanins, Blackcurrant	Increase *Lactobacilli,* *Bifidobacteria*; Decrease *Bacteroides* spp., *Clostridium* spp.	[90]
Flavanols, Cocoa	Increase *Bifidobacterial, Lactobacilli, E. rectale-C. coccoides*; Decrease *Clostridia*; While low–cocoa group: Increase *Clostridia, E. rectale-C. coccoides*	[91]
Proanthocyanins, Blueberry	Increase *Bifidobacterium, Prevotella* spp., *Bacteroides* spp., *Clostridium coccoides*; Decrease *Enterococcus* spp.	[92]
Flavonoid, Almond	Increase *Bifidobacterium* spp. and *Lactobacillus* spp.; Repress *pathogen Clostridum perfringens*	[93]
Red wine polyphenols	Increase *Bifidobacteria, Lactobacillus* and butyrate-producing (*Faecalibacterium prausnitzii* and *Roseburia*); Decrease Lipopolysaccharide (LPS)-producing (*Escherichia coli* and *Enterobacter cloacae*)	[94]
Proanthocyanidins, Cranberry	Increase abundance of *Bacteroidetes, Lachnospira* and *Anaerostipes*.; Decrease abundance of *Firmicutes,* *Clostridia, Oribacterium*	[95]
Catechins, Green tea	Increase *Firmicutes* and *Actinobacteria, Lachnospiraceae.*; Reduce *Bacteroidetes* and increase the FIR:BAC (*Firmicutes: Bacteroidetes*)	[96]
Red wine polyphenols	Increase the relative abundance of *Enterococcus, Prevotella, Bacteroides, Bifidobacterium, Bacteroides uniformis* groups	[97]
Anthocyanins, Tart cherry	High-Bacteroide: Increase *Lachnospiraceae, Ruminococcus, Collinsella*; Decrease *Bacteroides, Bifidobacterium*. Low-Bacteroides: Increase *Bacteroides* or *Prevotella* and *Bifidobacterium*; Decrease *Lachnospiraceae, Ruminococcus* and *Collinsella.*	[38]
Polyphenolic, *Schisandra chinensis*	Increase *Akkermansia, Roseburia, Bacteroides, Prevotella,* and *Bifidobacterium*	[98]
Increase	Increase *Clostridium, Lactobacillus, Faecalibacterium, Bifidobacterium*	[99]
Cocoa flavanols, Dark chocolate	Increase *Lactobacillus*; Decrease *Bacteroidetes*	[100]
Phenolic acids, Dietary raisin	Increase *Faecalibacterium prausnitzii, Bacteroidetes* spp., *Ruminococcus* spp.; Decrease *Klebsiella* spp., *Prevotella* spp., *Bifidobacterium* spp.	[101]
Apple polyphenol	Increase *Lactobacillus, Streptococcus*; Decrease *lecithinase-positive clostridia, Enterobacteriaceae, Pseudomonas*	[102]
Flavanones, Orange	Increase *Lactobacillus*; Decrease *Blautia coccoides*, *Clostridium leptum*	[103]
